# Watt-level continuous-wave antimonide laser diodes with high carrier-confined active region above 2.5 µm

**DOI:** 10.1186/s11671-024-03989-8

**Published:** 2024-03-12

**Authors:** Hongguang Yu, Chengao Yang, Yihang Chen, Tianfang Wang, Jianmei Shi, Juntian Cao, Zhengqi Geng, Zhiyuan Wang, Yu Zhang, Yingqiang Xu, Haiqiao Ni, Zhichuan Niu

**Affiliations:** 1 State Key Laboratory for Superlattices and Microstructures, Institute of Semiconductors, Chinese Academy of Sciences, Beijing, 100083 China; 2 Center of Materials Science and Optoelectronics Engineering, University of Chinese Academy of Sciences, Beijing, 100049 China

## Abstract

Thanks to high performance above room temperature, antimonide laser diodes have shown great potential for broad application in the mid-infrared spectral region. However, the laser`s performance noticeably deteriorates due to the reduction of carrier confinement with increased emission wavelength. In this paper, a novel active region with higher carrier confinements both of electron and hole, by the usage of an indirect bandgap material of Al_0.5_GaAs_0.04_Sb as the quantum barrier, was put up to address the poor carrier confinement of GaSb-based type-I multi-quantum-well (MQW) diode lasers emission wavelength above 2.5 µm. The carrier confinement and the differential gain in the designed active region are enhanced as a result of the first proposed usage of an indirect-gap semiconductor as the quantum barrier with larger band offsets in conduction and valence bands, leading to high internal quantum efficiency and low threshold current density of our lasers. More importantly, the watt-level output optical power is obtained at a low injection current compared to the state of the art. Our work demonstrates a direct and cost-effective solution to address the poor carrier confinement of the GaSb-based MQW lasers, thereby achieving high-power mid-infrared lasers.

## Introduction

In consideration of the numerous absorption lines for technological and greenhouse gases [1], light emitters operating in the mid-infrared spectral range are in great demand for numerous applications in the field of molecular spectroscopy [2], biomedical diagnostics [3], industrial process control [4], homeland security [5], and so on [6]. These emitters also attract much attention in infrared countermeasure [7], LIDAR [8], and free-space optical communications [9], in consequence of the high transparency atmospheric transmission window in this spectral range. Owing to compactness, scalability, high efficiency, and cost-effectiveness, the semiconductor laser stands as the predominant choice, presenting the most favorable Size-Weight and Power-Cost (SWaP-C) attribute compared to other laser types.

In comparison with other semiconductor materials and structure lasers, GaSb-based type-I multi-quantum-well (MQW) lasers have displayed critical competitiveness in the 1.9–3.3 µm range due to high efficiency, high working temperature and considerable continuous-wave output powers above room temperature [10, 11]. Nonetheless, the GaSb-based type-I MQW laser`s performance explicitly deteriorates with the increasing of emission wavelength on account of reduced carrier confinement, especially for hole confinement [12]. The poor hole confinement leads to an undesirable increase in the density of hole states in the active region including barrier states and waveguide states with higher volume to achieve the separation of electron and hole quasi-Fermi levels at high injection carrier concentration [13]. Worse still, the high threshold carrier concentration remarkably enlarges the non-radiative auger current, limiting the performance of the laser [14]. Additionally, the high concentration of holes leaking into the barrier and waveguide layers causes severe free carrier absorption and reduced injection carrier efficiency.

Previous studies on enhancing the hole confinement mainly focused on using the AlGaInAsSb quinternary alloy lattice-matched to GaSb as quantum barriers [15–17]. By adding indium and increasing the arsenic contents in the AlGaAsSb barrier layers, valence bands are pulled down, enlarging the valence band offsets of InGaAsSb/AlGaInAsSb quantum well. Nonetheless, it`s worthy to note that the growth of AlGaInAsSb is much complicated and difficult due to a large and severe thermodynamic miscibility gap [18]. The large differences in Al, Ga, and In atoms mobility and the sensitivity to growth parameters of the ratio of As and Sb in AlGaInAsSb bring many challenges in optimizing the growth of AlGaInAsSb. Although molecular beam epitaxy can achieve growth under far-form-equilibrium condition, the AlGaInAsSb grown is metastable and easy to phase separation [19], which severely restrict the performance of GaSb-based lasers. What is worse, a long time of growth interruptions is required to laboriously change the cell temperatures of Al, Ga, In, and needle valves of As and Sb to precisely control the composition of quinary and quaternary alloys, like AlGaInAsSb, AlGaAsSb, and InGaAsSb. This long time of growth interruptions not only increases the time cost of fabrication but also impacts the quality of interfaces between the quantum well and barrier. Therefore, it`s urgent to search for novel barrier materials or structures to enhance the carrier confinement in GaSb-based type-I MQW lasers. A simple and cost-effective approach is using high Al content of AlGaAsSb as the quantum barrier to address the poor confinement of GaSb-based type-I QW. The AlGaAsSb matched to GaSb is stable and easy to grow in a large growth window. Obviously, the band offsets in conduction and valence bands would be enlarged with the composition of Al further increasing, regardless of whether it is a direct bandgap material or an indirect bandgap material [20]. But there are few studies on the use of indirect bandgap semiconductors as barriers to enhance carrier confinement in GaSb-based quantum-well lasers. Although the GaAs/AlGaAs quantum well has been extensively studied [21, 22], which pointed out that intervalley scattering and Γ-, X-, and L-band would affect the carrier transport characteristics when the AlGaAs changes from direct-gap to indirect-gap material, it is unclear what impact the use of an indirect-gap semiconductor as barriers in quantum-well lasers on laser performance in experiments.

In this research, for the first time, the quaternary alloy of Al_0.5_GaAs_0.04_Sb, an indirect-gap semiconductor was used as the quantum barrier to improve the confinements both of electrons and holes and the In_0.44_GaAs_0.18_Sb with a compressive strain of 1.5% was used as the quantum well to achieve the lasing wavelength of around 2.5 µm. This designed active region of In_0.44_GaAs_0.18_Sb/Al_0.5_GaAs_0.04_Sb quantum well with large band offsets in conduction and valence bands not only prevents carrier leakage but also reduces the threshold carrier density and enhances the differential gain of this active region. More specifically, the high quantum well depth can further improve the energy spacings between the first two conduction and valence subbands, and also allow to use of the high indium composition of InGaAsSb as quantum well with smaller carrier mass associated with low carrier density of states [23]. These features can lower the threshold carrier density and improve the differential gain by minimizing the number of states in the conduction and valence bands that are available for the population [24]. Based on theoretical calculations of the variation in optical gain with injected carrier concentration within the active region and specific experimental data of our growth and fabricated GaSb-based type-I MQW laser, this novel active region is demonstrated to possess low threshold and high differential gain characteristics. Furthermore, the watt-level output power is obtained at low injection current compared to the state of the art. Our work demonstrates a direct and simple alternative approach to achieving high-power mid-infrared light emitters.

## Design and experiments

**Fig. 1:**
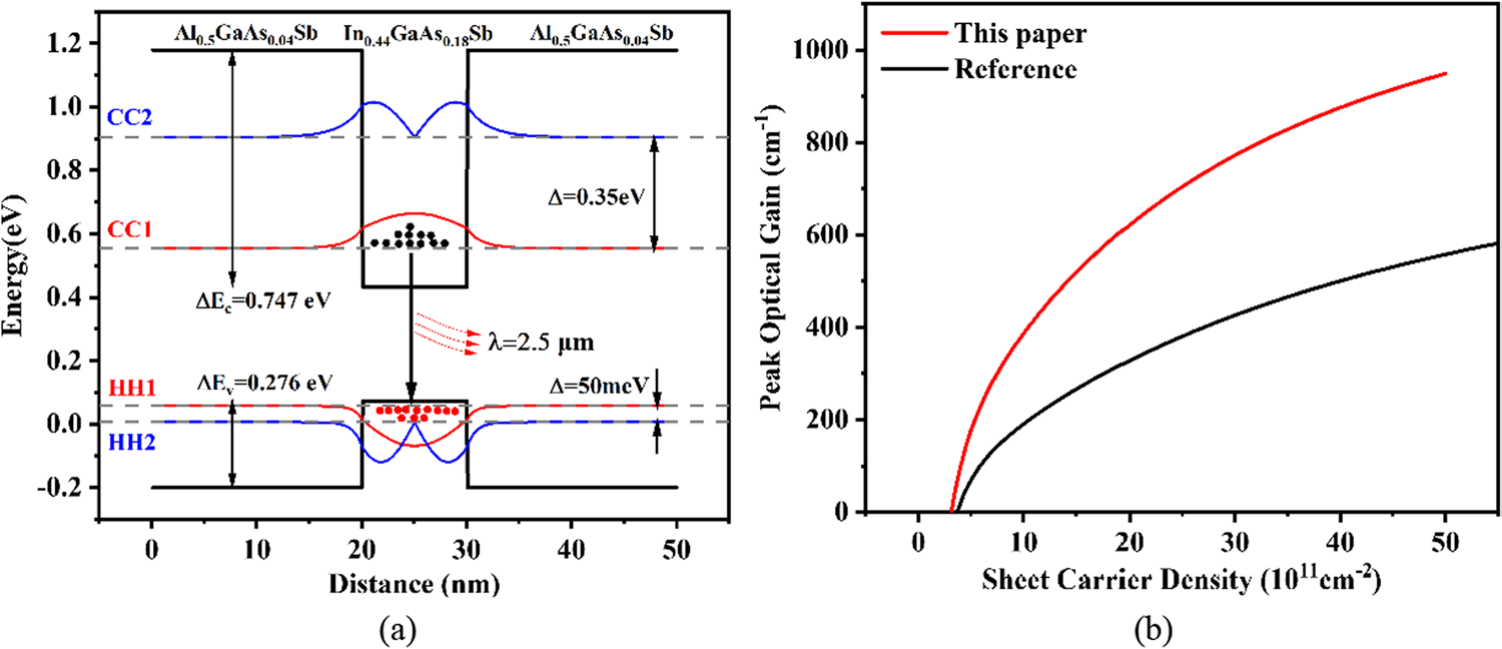
**a** The quantum well structure of 20-nm Al_0.5_GaAs_0.04_Sb/10-nm In_0.44_GaAs_0.18_Sb. The dashed lines are the results of conduction and valence subbands. **b** the peak optical gain versus injection sheet carrier density of this paper and reference [29]

In this paper, the 20 nm-wide Al_0.5_GaAs_0.04_Sb matched with GaSb is used as the quantum barrier layer, and the 10 nm-wide In_0.44_GaAs_0.18_Sb with 1.5% compressive strain is regarded as the quantum well layer to achieve the lasing wavelength of around 2.5 µm. Figure 1 shows the band structure at the Gamma point, calculated based on the 8-band k · p model [25], and the optical gain spectrum calculated based on Fermi`s Golden Rule [26]. The material data as input parameters for calculation are taken from the review [27] and paper [28]. Figure 1a demonstrates that large conduction and valence band offsets result in more subbands in the quantum wells, preventing pinning of the quasi-Fermi level too early, especially for the hole quasi-Fermi level. That means there are sufficient electron and hole states in QW to achieve population inversion, avoiding carrier leaking into the barrier and waveguide layers. Furthermore, the large band offsets also enlarge the energy spacing between the conduction subbands and valence subbands. As Fig. 1a shows, the energy spacing between the first and second conduction subbands (CC1 and CC2) is 350 meV, and as for valence subbands (HH1 and HH2), it is 50 meV. These high energy spacings force the electrons and holes to occupy the CC1 and HH1 as much as possible to achieve lasing, lowering the threshold carrier density. Figure 1b presents peak optical gain versus injection sheet carrier density, which explicitly proves that the active region of this paper has a much higher differential gain. The details of the calculation method for the gain vs carrier density were clearly described in [23]. And the active region of the reference device [29] is a 14.5-nm In_0.41_GaAs_0.14_Sb quantum well with a 200-nm Al_0.25_GaAs_0.02_Sb quantum barrier.

**Fig. 2:**
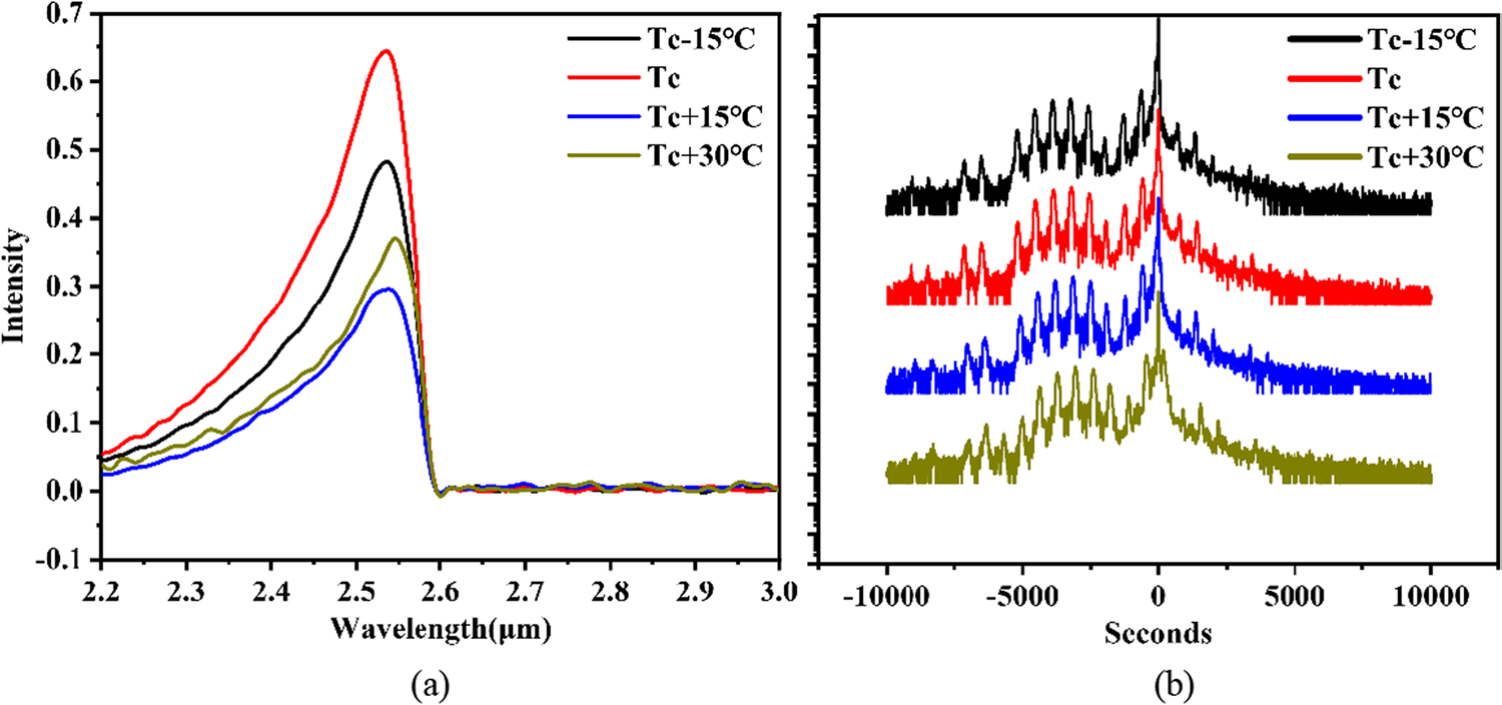
The four samples of Al_0.5_GaAsSb/In_0.44_GaAs_0.18_Sb MQWs grown in different temperatures. **a** The room-temperature photoluminescence spectra, **b** the HR-XRD curves

Epitaxial growth was performed on N-type GaSb (1 0 0) substrates by solid-source Gen-II molecular-beam epitaxy equipped with valved cracked cells for providing As_2_ and Sb_2_. Four multi-quantum-well samples were grown at different temperatures respectively Tc-15 °C, Tc, Tc + 15 °C, and Tc + 30 °C in which the Tc is the reconstruction phase transitions temperature of GaSb (2 × 5 → 1 × 3) based on reflective high energy electron diffraction (RHEED). As Fig. 2a shows, the sample of Tc has the maximum photoluminescence intensity at the same test condition, although there is no obvious difference between these HR-XRD curves in Fig. 2b. The photoluminescence spectra are a more sensitive and elaborate characterization method than high-resolution X-ray diffraction and atomic force microscope to evaluate the crystal quality of samples, especially for optical materials [30]. Thus, the Tc was chosen as the optimal growth temperature of the active region.

**Fig. 3:**
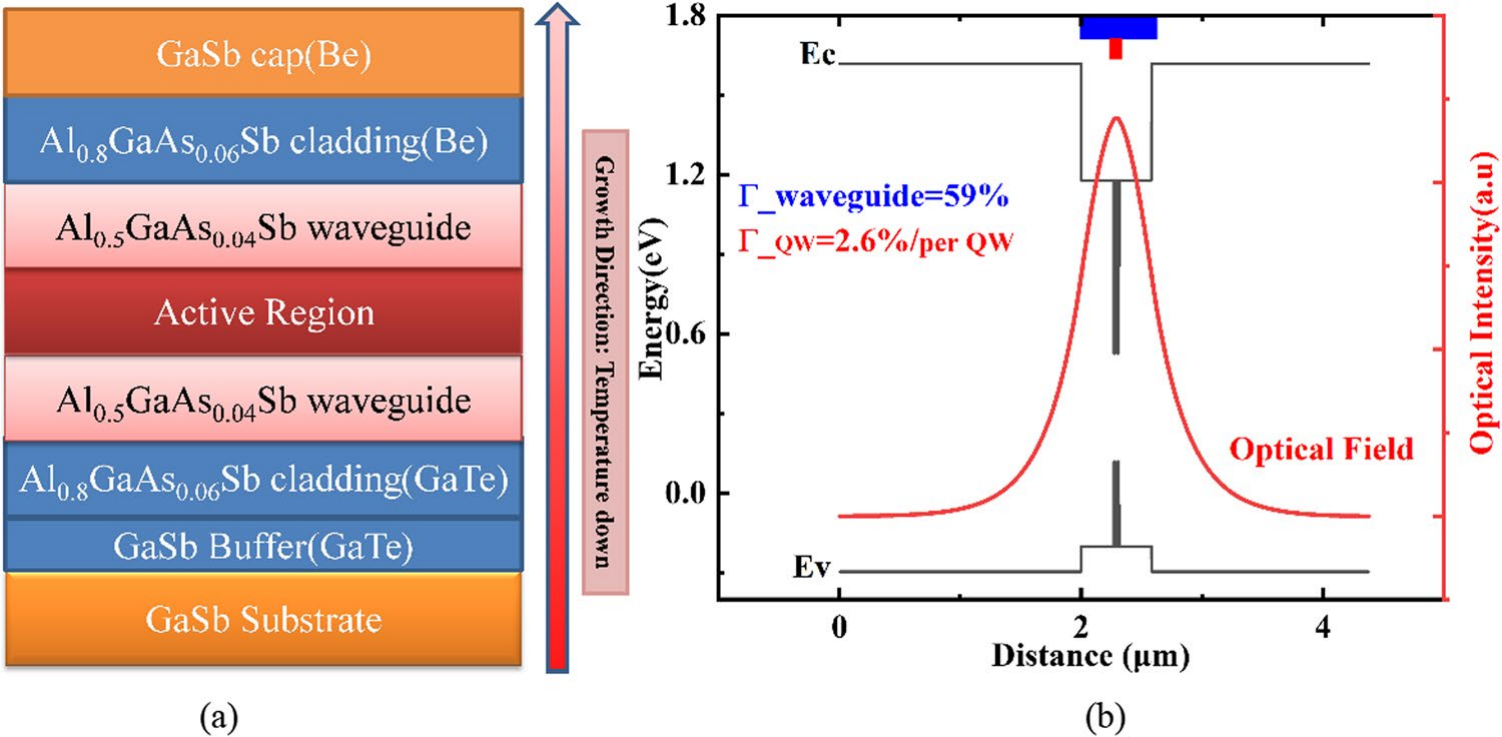
**a** The whole epitaxial structure of 2.5 µm GaSb-based diode laser. **b** The energy band and the optical near field of fundamental mode distribution in the growth direction

Figure 3 depicts the full structure of the laser and the band diagram with the near-field optical fundamental mode distribution. A 366 nm heavily n-type GaSb buffer, enhancing the flatness and electroconductivity, was firstly grown on 2-inch Te doped n-GaSb substrates. Two 10-nm thick 1.5% compressive strained In_0.44_GaAs_0.18_Sb QWs spaced by 20-nm thick lattice matched Al_0.5_GaAs_0.04_Sb barrier layers were regarded as the active region. The 580-nm waveguide core including the active region and separate confinement layers is sandwiched between 2- and 1.8-µm wide Al_0.8_GaAs_0.06_Sb cladding layers with 3.3 × 10^17^ cm^−3^ *n*-type and 6.5 × 10^18^ cm^−3^ *p*-type doping, respectively. It’s worth noting that the indirect-gap material of Al_0.5_GaAs_0.04_Sb is also used as the undoped waveguide layer, decreasing the time of growth interruptions to laboriously change the cell temperatures of the Al source cell. Note that after the active region grown over, the rest of the epitaxial layers were grown at the same temperature as the active region to avoid the optical and crystal quality of the active region deteriorating with annealing [31].

The grown wafer was processed into 100-µm-wide FP cavity lasers with different cavity lengths, using standard contact optical lithography in combination with the etching process. A 250-nm thick SiO_2_ insulation layer was deposited via plasma enhanced chemical vapor deposition. The low-resistance ohmic contacts on the p-type and n-type sides were achieved by magnetron sputtered Ti/Pt/Au and AuGeNi. Finally, a single emitter was chipped and p-side down to a copper heatsink, ready to test its performance. The output power was measured using a Coherent PowerMax PM10 with FieldMaxII.

## Results and discussion

In order to better compare the performance of our diode laser with state-of-art laser. It`s necessary to provide a brief overview of the advanced characteristics of GaSb-based type-I MQW lasers emitting around 2.5 µm. The most advanced CW output characteristics were reported in 2002 [29], which shows the maximum CW power is 1 W with power conversion efficiency of about 8% at an injection current of 7 A from a 100-µm-wide and 2-mm-long chip at 12 °C with the threshold current density of 250 A/cm^2^, the low series resistance of 0.1 Ω, the internal losses of 4 cm^−1^, and the internal quantum efficiency of 0.5. In our work, we guarantee both the high-power output of diode lasers emitting at approximately 2.5 µm and the low threshold characteristics inherent in our designed laser, by the usage of an indirect-gap semiconductor as the quantum barrier, as demonstrated and discussed in the following sections.

**Fig. 4:**
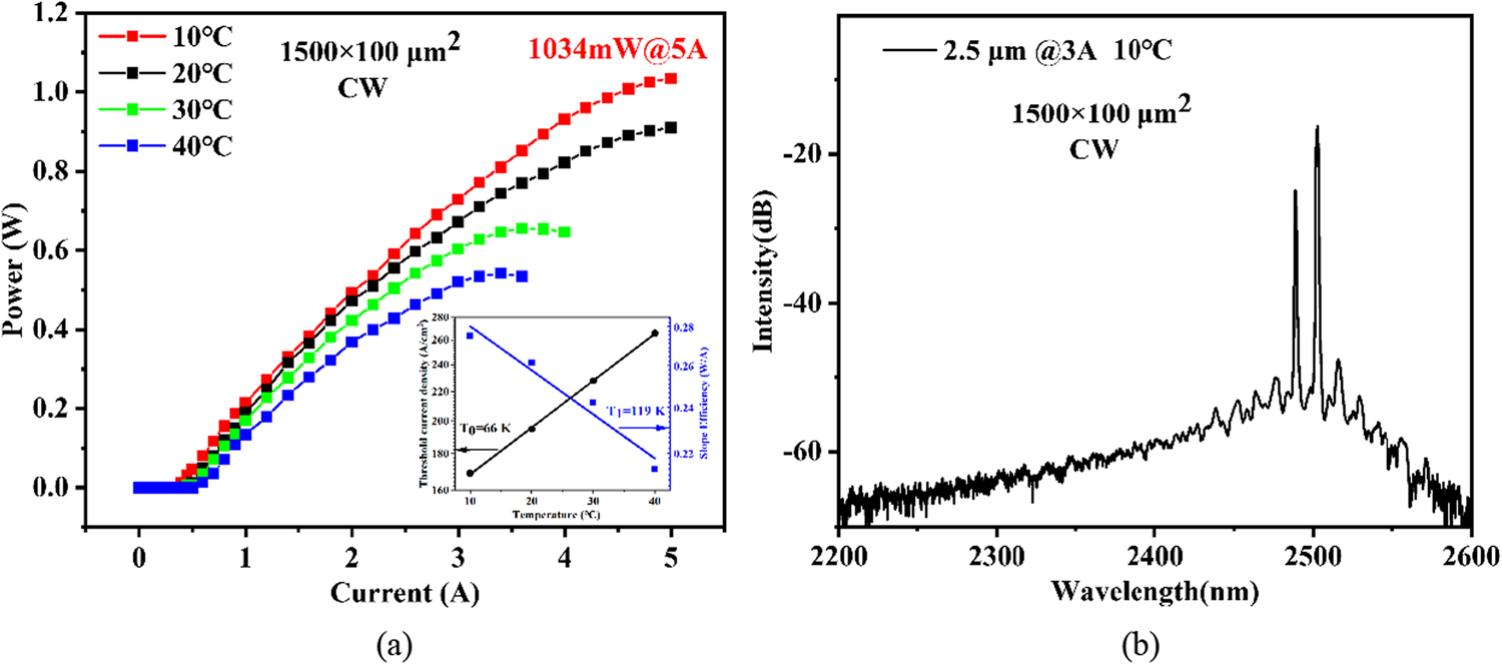
**a** Continuous-wave output characteristics of 1.5-mm-long and 100-µm-wide laser at 10–40 °C. The inset shows characteristic temperatures T_0_ and T_1_. **b** The CW emission spectra at 3 A and 10 °C

**Fig. 5:**
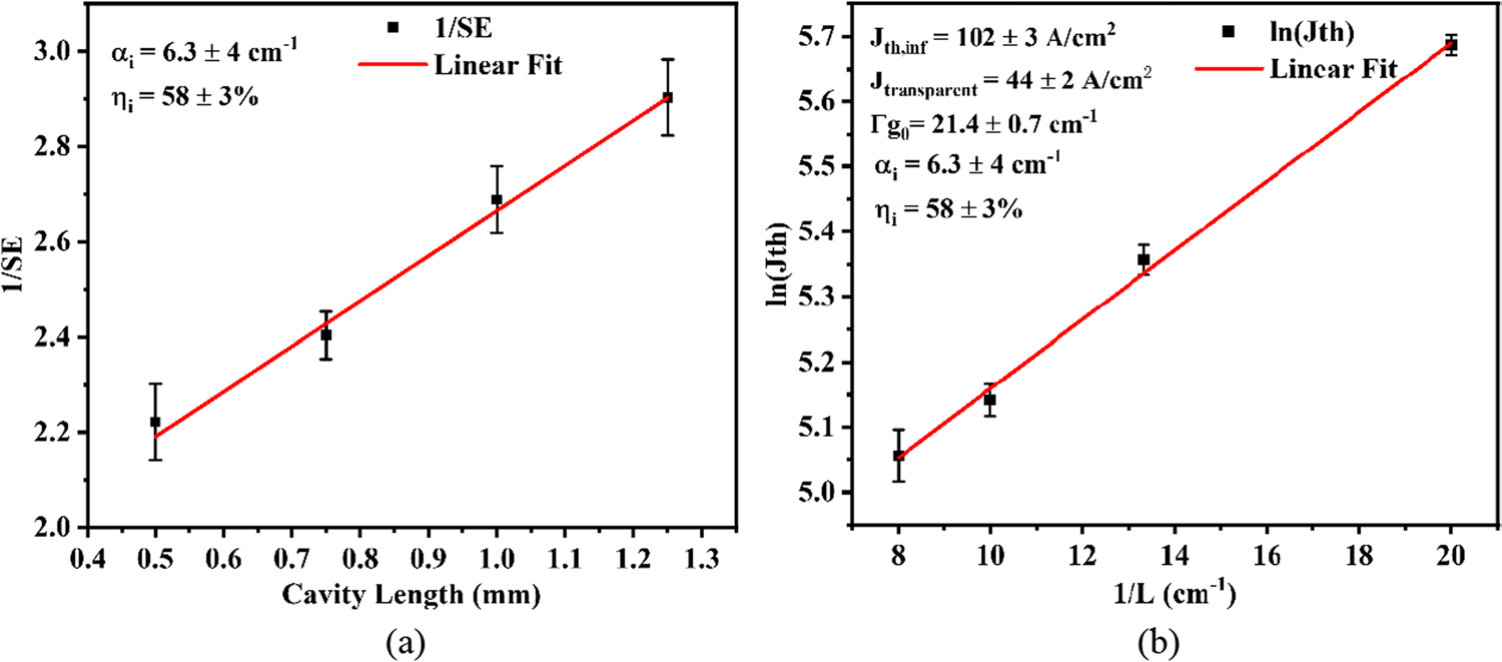
**a** The linear regression between the reciprocal of the slope efficiency and the cavity length. **b** The linear regression between the logarithm of the threshold current density and the reciprocal of the cavity length. The short vertical lines stand for the error of measured threshold current density. And the uncertainties in the derived quantities are also displayed

The continuous-wave output power (P–I) characteristics of 1.5-mm-long and 100-μm-wide uncoated diode lasers, measured at heatsink temperatures from 10 to 40 °C, are displayed in Fig. 4a. The inset figure shows the characteristic temperatures T_0_ and T_1_ are respectively 66 K and 119 K. A maximum power of 1034 mW is obtained at 5 A under heatsink temperature of 10 °C, comparable to the state-of-art performance. And the laser displays a low threshold current density of 170 A/cm^2^ with a slope efficiency of about 0.275 W/A. Also, the emission spectrum is obtained with a Yokogawa AQ6376 optical spectrum analyzer with a multimode index fiber. Figure 4b shows the laser spectra at 3 A and 10 °C. The internal loss and the internal quantum efficiency are calculated based on the linear regression between the reciprocal of the slope efficiency and the cavity length in Fig. 5a. The internal quantum efficiency of 0.58 surpasses the data of the most high-performing GaSb-based laser reported emitting around 2.5 µm, which exhibits an internal quantum efficiency of 0.5 [29]. Furthermore, the threshold current density at infinite cavity length, the transparent current density, and the effective gain coefficient are extrapolated by the following equations.

The threshold current density can be expressed as

1 $${J}_{th}=\frac{{J}_{tr}}{{\eta }_{i}}{e}^{({\alpha }_{i}+{\alpha }_{m})/\Gamma {g}_{0}}$$

where ${J}_{tr}$ is the transparent current density, ${\eta }_{i}$ is the internal quantum efficiency, ${\alpha }_{i}$ and ${\alpha }_{m}$ are respectively the internal loss and the mirror loss, $\Gamma$ is the confinement factor of quantum wells, and ${g}_{0}$ is the gain coefficient near threshold. The mirror loss ${\alpha }_{m}$ is given as

2 $${\alpha }_{m}=\frac{{\text{ln}}(1/{R}_{1}{R}_{2})}{2L}$$

By playing logarithmic transformation of both sides, Eq. (1) can be written as

3 $${\text{ln}}\left({J}_{th}\right)={\text{ln}}\left(\frac{{J}_{tr}}{{\eta }_{i}}\right)+\frac{{\alpha }_{i}}{\Gamma {g}_{0}}+\frac{{\text{ln}}(1/{R}_{1}{R}_{2})}{2\Gamma {g}_{0}}\frac{1}{L}$$

When the cavity length is infinite ($L\to \infty$), the threshold density is

4 $${J}_{th,inf}=\frac{{J}_{tr}}{{\eta }_{i}}{e}^{{\alpha }_{i}/\Gamma {g}_{0}}$$

So, the effective gain coefficient $\Gamma {g}_{0}$ of 21.4 cm^−1^, the transparent current density ${J}_{tr}$ of 44 A/cm^2^, and the threshold current density at infinite cavity length ${J}_{th,inf}$ of 102 A/cm^2^ are obtained by implementing the linear regression between the logarithm of the threshold current density and the reciprocal of cavity length, as demonstrated in Fig. 5b. The transparent current density of 44 A/cm^2^ is much improved than those of antimonide lasers [32], which proves that the active region designed in this paper has a higher differential gain.

**Fig. 6:**
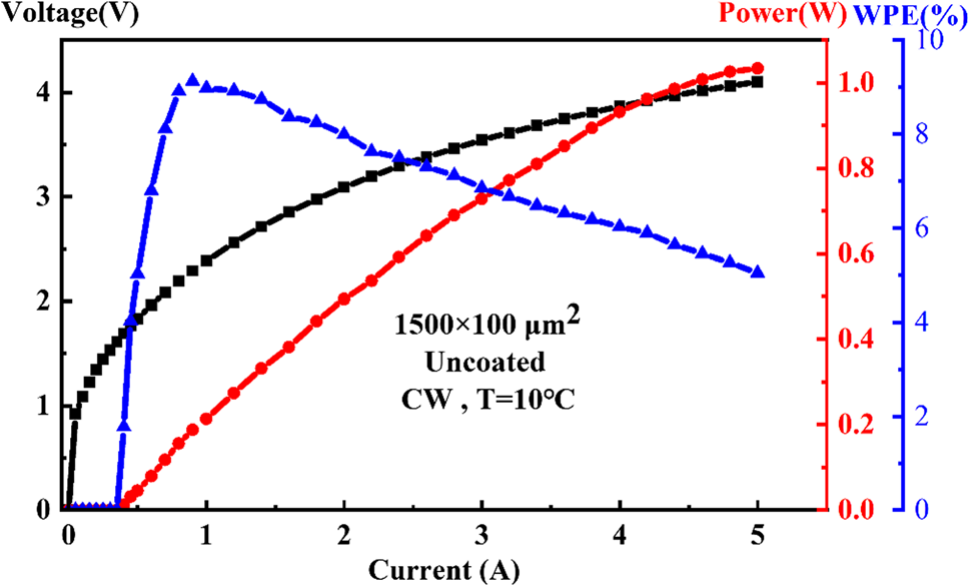
The continuous-wave output characteristics of 1.5-mm-long and 100-µm-wide uncoated diode lasers at 10 °C

For diode lasers, the wall-plug efficiency (WPE) of a single chip is important performance in practical applications. The continuous-wave characteristics of 1.5-mm-long and 100-μm-wide uncoated diode lasers at 10 °C, are shown in Fig. 6. The power conversion efficiency at the maximum output power is relatively low due to the relatively high operated voltage of our lasers. However, the watt-level output power is obtained at a low injection current of 5A compared to the state of the art, even under conditions of relatively high operating voltage and series resistance, which associates with a high heat production capability, preventing a further increase in the CW output power of our laser. In consideration of the first demonstration of this technology, the output power of our lasers would increase obviously with an optimized design to operate with lower voltage and series resistance. In a word, the usage of an indirect-gap semiconductor as the quantum barrier indeed improves the carrier confinement and the differential gain in GaSb-based type-I MQW active region, not only maintains a state-of-the-art high output power but also shows great potential in the future to provide a novel and cost-effective solution to address the issue of insufficient carrier confinement of the GaSb-based MQW lasers, thereby achieving high-power output in mid-infrared lasers.

## Conclusions

In summary, we have developed high-power 2.5 µm GaSb-based diode laser with a low threshold by designing a novel active region with enhancements in carrier confinement with the usage of an indirect-gap semiconductor as the quantum barrier. The improvements in both electrons and holes confinement lead to an ultralow transparent current density of 44 A/cm^2^ and high internal quantum efficiency of 0.58. And the watt-class continuous-wave power was obtained with the low threshold current density of 85 A/cm^2^ per quantum well and slope efficiency of 0.275 W/A in low injection current. We prove that the usage of an indirect-gap semiconductor as the barrier can improves the carrier confinement and the differential gain of the InGaAsSb/AlGaAsSb quantum-well lasers, upgrading the output performance of GaSb-based type-I lasers, thereby achieving high-power output in mid-infrared lasers.

## Data Availability

The datasets used and analyzed during the current study are available from the corresponding author upon reasonable request.
